# Differences in Health-Related Quality of Life in Older People with and without Sarcopenia Covered by Long-Term Care Insurance

**DOI:** 10.3390/ejihpe12060040

**Published:** 2022-05-26

**Authors:** Masahiro Kitamura, Kazuhiro P. Izawa, Kodai Ishihara, Peter H. Brubaker, Hiroaki Matsuda, Soichiro Okamura, Koji Fujioka

**Affiliations:** 1 School of Physical Therapy, Faculty of Rehabilitation, Reiwa Health Sciences University, 2-1-12 Wajirogaoka, Fukuoka 811-0213, Japan; pt_masa0808@yahoo.co.jp; 2 Department of Public Health, Graduate School of Health Sciences, Kobe University, 7-10-2 Tomogaoka, Kobe 654-0142, Japan; mhe1601@std.huhs.ac.jp; 3 Cardiovascular Stroke Renal Project (CRP), Kobe 654-0142, Japan; brubaker@wfu.edu; 4 Department of Rehabilitation, Sakakibara Heart Institute of Okayama, 5-1 Nakaicho 2-chome, Okayama 700-0804, Japan; 5 Department of Health and Exercise Science, Wake Forest University, Winston-Salem, NC 27106, USA; 6 Department of Rehabilitation, Rifuru Yukuhashi Day Care Center, 379-1 Takase, Yukuhashi 824-0027, Japan; matmat1213@gmail.com (H.M.); okamura19830216@gmail.com (S.O.); kouji.0314yh@gmail.com (K.F.)

**Keywords:** quality of life, sarcopenia, long-term care insurance, older people, physical function, handgrip strength, one-leg standing time, gait speed, daycare center, Japan

## Abstract

Background: As a result of the increase in older people covered by long-term care insurance (LTCI), prevention of sarcopenia and maintenance and improvement of health-related quality of life (HRQOL) have become important themes. This study aimed to clarify both the differences in HRQOL in older people with and without sarcopenia covered by LTCI and the correlation between HRQOL and physical function. Methods: Participants were 101 older people with LTCI at a daycare center in Japan. We investigated clinical factors using the EuroQol five-dimension three-level questionnaire (EQ-5D-3L). Analysis was by unpaired *t*-test, Mann–Whitney U test, chi-square test, analysis of covariance, Pearson’s correlation coefficient, and Spearman’s rank correlation coefficient. Results: Compared to the no sarcopenia group (*n* = 40), the sarcopenia group (*n* = 24) had significantly lower body mass index, skeletal muscle mass index, gait speed, EQ-5D-3L, and adjusted EQ-5D-3L (*p* < 0.05). The EQ-5D-3L showed a significant correlation with handgrip strength in the sarcopenia group (*p* = 0.02) and significant correlations with gait speed and one-leg standing time (both, *p* = 0.01) in the no sarcopenia group. Conclusion: We clarified differences in HRQOL in older people with and without sarcopenia covered by LTCI. This information on the interrelationship between HRQOL and physical function may help maintain and improve HRQOL in these people.

## 1. Introduction

The number of older people is increasing rapidly worldwide [1], and Japan, in particular, is the most rapidly aging country globally [2]. In Japan, people over the age of 65 accounted for 28.8% of the total population in 2020 and are projected to account for approximately 40% by 2050 [2]. The Long-Term Care Insurance System (LTCI) was launched in 2000 with the aim of socially supporting the need for long-term care and reducing the burden on families who provide such care in Japan [3,4,5]. The LTCI is aimed at people aged 65 and over, and people aged 40 to 64 with specific illnesses, and certification of care is determined on seven levels (support levels 1–2 and care levels 1–5) depending on their needs [5]. Care levels require more care than support levels, and a higher level indicates a higher need for care. The number of LTCI users has tripled in 20 years, from 2.18 million in 2000 to 6.9 million in 2021, and the cost of LTCI is a serious problem [6,7,8].

People with LTCI have higher rates of severity and mortality than those without LTCI, and the presence of sarcopenia is a known risk factor [9,10]. Sarcopenia is defined as the presence of decreased skeletal muscle mass and decreased muscle strength and physical function with aging, and diagnostic criteria have been established [11]. The prevalence of sarcopenia is as high as 5–19% [10,12,13] in older people without LTCI and 48% in older people with LTCI [14]. Having sarcopenia has also been shown to be associated with fall and fracture events, decreased activities of daily living (ADL), increased mortality, and increased costs for medical and long-term care insurance [15,16,17]. Moreover, older people with LTCI who have sarcopenia are reported to have low physical activity and are presumed to be at very high risk [18]. Therefore, it is important to evaluate and take measures against sarcopenia to prevent its aggravation.

Maintaining a high quality of life is one of the main goals in the care of older people [19]. Health-related quality of life (HRQOL) is used to assess quality of life is associated with physical function, psychological aspects, and falls [20]. As the number of older people increases, their risks increase, and thus, the importance of HRQOL is also increasing [21]. In particular, efforts to improve HRQOL are attracting attention as a countermeasure to the problems of older people with LTCI [22]. Many reports on HRQOL in older people have revealed that it is related to physical functions such as handgrip strength and gait speed, in addition to mental status, social and environmental factors, and prognosis [23].

However, the differences in HRQOL between those with and without sarcopenia among older people covered by LTCI were unclear in previous studies. Furthermore, the interrelationship between each HRQOL measure and physical function was not clear in this particular age group. We hypothesized that among older people with LTCI, those with sarcopenia would have lower HRQOL than those with no sarcopenia. Therefore, the purposes of this study were to clarify both the differences in HRQOL in older people with and without sarcopenia covered by LTCI, and the correlation between HRQOL and physical function in those with and without sarcopenia.

### Relationships Investigated by this Study

- Prevalence of sarcopenia among older people with LTCI.
- Differences in physical function and HRQOL in older people with and without sarcopenia covered by LTCI.
- Correlation between HRQOL and physical function in older people with sarcopenia covered by LTCI.
- Correlation between HRQOL and physical function in older people without sarcopenia covered by LTCI.

## 2. Related Work

### 2.1. Relationship between Sarcopenia and HRQOL in Older People

A previous case-control study reported that older people with sarcopenia had lower physical function, HRQOL, and nutritional status than those without sarcopenia [24]. In one cross-sectional study, compared to those without sarcopenia, older males with sarcopenia had lower income, physical function, BMI, and HRQOL, and older females with sarcopenia had higher BMI and lower HRQOL [25]. Another cross-sectional study comparing severe and moderate sarcopenia with non-sarcopenia groups showed that the severe and moderate sarcopenia groups were characterized by older women, higher rates of unemployment, lower ADL, and lower HRQOL. In addition, severe sarcopenia has been shown to be associated with HRQOL [26]. A cohort study using sarcopenia screening tools reported that older people at high risk for sarcopenia had lower physical performance, cognitive function, and HRQOL than older people at low risk for sarcopenia [27]. A systematic review of sarcopenia and HRQOL reported that sarcopenia and HRQOL are associated in the elderly [28]. Therefore, older people with sarcopenia may be associated with lower HRQOL.

### 2.2. Sarcopenia in Older People with LTCI

A cross-sectional study comparing a sarcopenia group with a no sarcopenia group reported low physical activity in the older people with LTCI in the sarcopenia group [18]. Low physical activity in the sarcopenia group was also reported to be associated with LTCI utilization [29]. In addition, age and BMI are risk factors for sarcopenia in older people with LTCI [30], and it was reported that onset of sarcopenia and low nutritional status may increase incident disability in LTCI [10,31]. Therefore, sarcopenia in the elderly with LTCI may be associated with low physical activity and poor nutrition, and the presence of sarcopenia may raise the cost of LTCI due to additional incident disability. However, the number of reviews was small, and no articles related to HRQOL in older people covered by LTCI were found.

### 2.3. HRQOL in Older People with LTCI

In older people with LTCI, HRQOL is higher in women than in men, more people live alone than with other families, and increased levels of LTCI care/support are associated with decreased HRQOL [32]. In addition, ADL in older people with LTCI was shown to correlate weakly with HRQOL [33]. Decreased HRQOL in older people is also associated with LTCI use and mortality risk [34]. Thus, the HRQOL of older people with LTCI may be associated with gender differences, number of people living together, LTCI levels, ADL, and risk of death. However, the number of articles was extremely small, and no articles related to sarcopenia were found that investigated the HRQOL of older people covered by LTCI. Therefore, we need to consider HRQOL of older people with and without sarcopenia who are covered by LTCI.

## 3. Materials and Methods

### 3.1. Study Population

In this cross-sectional study, we investigated the records of 101 consecutive older people with LTCI who underwent rehabilitation at one daycare center in Japan from November 2018 to June 2019. We included participants over the age of 65 who were able to walk with or without assistive devices and excluded participants in whom skeletal muscle mass index (SMI) could not be measured and those with severe dementia. This study was approved by the Fukuokawajiro Rehabilitation Training College Ethics Committee (approval No. FW-20-01), and each participant gave informed consent in writing.

### 3.2. Clinical Characteristics of the Participants

The participants’ characteristics were retrospectively assessed from their clinical records by two physical therapists. Characteristics evaluated included age, sex, body mass index (BMI), SMI, LTCI level, and comorbidities, and, as indices of HRQOL, handgrip strength, one-leg standing time, gait speed, and EQ-5D-3L (EuroQol 5-dimension 3-level) were used.

### 3.3. SMI and Physical Function Evaluation

SMI was evaluated using a multi-frequency electrical impedance meter (InBody 430, Biospace Japan, Tokyo, Japan) [35]. We used the value obtained by dividing the skeletal muscle mass of the left and right limbs by the square of the height. A Smedley-type hand dynamometer (TKK5401, Takei Equipment Industry Co., Ltd., Niigata, Japan) was used to evaluate handgrip strength [36]. We measured twice on both sides and used the maximum value obtained. Gait speed was measured using a stopwatch and was defined as the time required to walk 6 m at normal speed [37]. To measure one-leg standing time, we used a stopwatch to measure the longest time each of the participants held this posture with their eyes open. The measurement was performed twice on the left and right legs, and the maximum value was set to 60 s [37].

### 3.4. Sarcopenia

Sarcopenia was determined according to the criteria of the Asian Working Group for Sarcopenia [11]. The criteria indicating sarcopenia are a handgrip strength of 28.0 kg or less for males and 18.0 kg or less for females; a normal gait speed of 1.0 m/s or less; and a SMI of 7 kg/m^2^ or less for males and 5.7 kg/m^2^ or less for females [11].

### 3.5. HRQOL

The Japanese version of the EQ-5D-3L was used for the HRQOL evaluation [38]. This EQ-5D-3L scale contains five dimensions that rate “mobility”, “self-care”, “usual activities”, “pain/discomfort”, and “anxiety/depression”. Each dimension has three levels: “no problem”, “some problem”, and “extreme problem”, and the EQ-5D-3L can distinguish between 243 health conditions. The collected EQ-5D-3L scales were converted to a global utility score using the Japanese version of the time trade-off value set, with a higher score indicating better HRQOL [39].

### 3.6. LTCI Levels

The LTCI system in Japan was introduced to meet the demands of older people based on a user-type social insurance system that supports independence [5]. The LTCI level is determined by the LTCI committee in the area where the person lives. The targets are people aged 65 years and over, and those aged 40 to 64 years with certain illnesses. This study did not include people aged 40–64 years with LTCI. People who are independent in ADL but need to monitor some of their ADL, such as shopping, are at support Level 1, and those with reduced gait ability due to lower limb muscle weakness are at support Level 2. Those who need simple care as part of their ADL are at Care Level 1. Those who need some self-care of ADL, such as for eating, excretion, and bathing, are at care Level 2. Care Level 3 is for those who walk with assistive devices or use a wheelchair to move and require care for many ADL. Those who use a wheelchair to move and cannot perform ADL without care are at care Level 4. Finally, those who are bedridden, have difficulty communicating, and cannot eat alone are at care Level 5. These LTCI levels were determined from the participants’ clinical data by two physiotherapists.

### 3.7. Statistical Analysis

Regarding the notation of participant characteristics and clinical parameter values, categorical variables are shown as a percentage and continuous variables are shown as the mean ± SD. The Shapiro–Wilk test was used for variable normality. To compare between two groups of participant characteristics and clinical parameters, the unpaired *t*-test was used for continuous variables with a normal distribution, the Mann–Whitney *U* test for continuous variables without a normal distribution, and the chi-square test for categorical variables. The mathematical expression of each statistical method is as the follows.

- Unpaired *t*-test:

$$\begin{array}{c} t=\frac{M_{1}-M_{2}}{\sqrt{\frac{n_{1}a_{1}^{2}+n_{2}a_{2}^{2}}{n_{1}+n_{2}-2}\left(\frac{1}{n_{1}}+\frac{1}{n_{2}}\right)}}\;\;df=n_{1}+n_{2}-2\\ n=sample\;size,\;a^{2}=variance,\;M=average \end{array}$$

- Mann–Whitney *U* test:

$$\begin{array}{c} U=min\left(R_{1}-\frac{n_{1}\left(n_{1}+1\right)}{2},\;R_{2}-\frac{n_{2}\left(n_{2}+1\right)}{2}\right)\;\;E\left[U\right]=\frac{n_{1}n_{2}}{2}\\ a\left[U\right]=\sqrt{\frac{n_{1}n_{2}\left(n_{1}+n_{2}+1\right)}{12}}\;\;z=\frac{U-E\left[U\right]}{\sqrt{a\left[U\right]}}\\ n=sample\;size,\;\;R=Sum\;of\;ranks,\;\;a=variance \end{array}$$

- Chi-square test:

$$\chi^{2}=\sum \frac{{\left(observed-expected\right)}^{2}}{expected}$$

Analysis of covariance was used to compare the differences in HRQOL between the two groups. The covariates used were variables that showed a significant difference between the two groups, age, and sex, excluding factors related to sarcopenia criteria. The analysis of covariance was calculated as:

$$\begin{array}{c} y_{ij}=\mu +\tau_{i}+slope\left({\bar{x}}_{ij}-x\right)+\varepsilon_{ij}\\ \mu =total\;average\;of\;outcomes,\;\;\tau =effect\;of\;factors,\;\;\varepsilon =error\; \end{array}$$

Pearson’s correlation coefficient and Spearman’s rank correlation coefficient were used to determine the correlation between HRQOL and body composition and physical function in each of the sarcopenia and no sarcopenia groups. The mathematical expression for Pearson’s correlation coefficient and Spearman’s rank correlation coefficient were, respectively:

$$=\frac{\sum_{i=1}^{n}\left(x_{i}-\bar{x}\right)\left(y_{i}-\bar{y}\right)}{\sqrt{\sum_{i=1}^{n}{\left(x_{i}-\bar{x}\right)}^{2}\sqrt{\sum_{i=1}^{n}{\left(y_{1}-\bar{y}\right)}^{2}}}}\;\;\;n=sample\;size$$

and:

$$\begin{array}{c} r=1-\frac{6\sum d^{2}}{N^{3}-N}\;.\\ N=number\;of\;x\;and\;y\;pairs,\;\;d=difference\;in\;rank\;between\;x\;and\;y\;values \end{array}$$

The correlation coefficient was interpreted as follows: <0.2 indicated “no correlation”, 0.2 to 0.4 “low correlation”, 0.4 to 0.7 “moderate correlation”, 0.7 to 0.9 “high correlation”, and 0.9 or more “very high correlation” [40]. A *p*-value of <0.05 was considered to indicate statistical significance. Statistical analyses were performed with IBM SPSS 25.0 J statistical software (IBM SPSS Japan, Inc., Tokyo, Japan).

## 4. Results

### 4.1. Participant Flow

**Figure 1:**
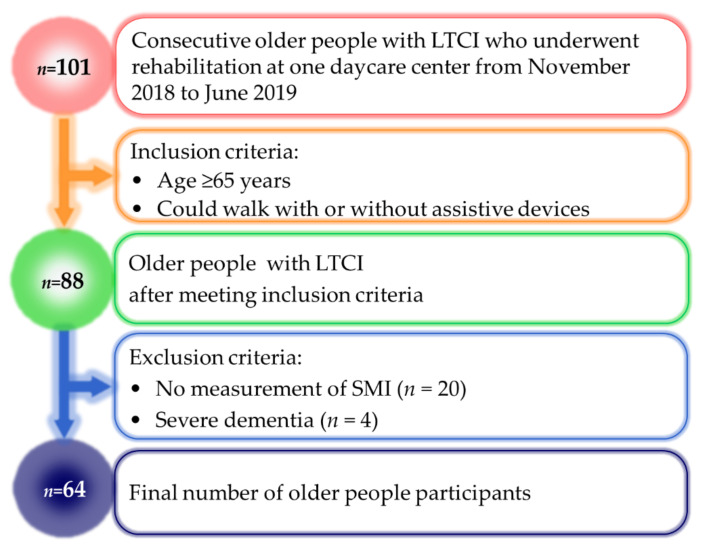
Participant flow. LTCI, long-term care insurance; SMI, skeletal muscle mass index.

Figure 1 shows the flow of participants in this study. The study initially included 88 of 101 consecutive older people with LTCI who underwent rehabilitation and met the inclusion criteria. We excluded 20 older people in whom SMI could not be measured and four older people with severe dementia. The remaining 64 older people were divided into the sarcopenia group (*n* = 24) and no sarcopenia group (*n* = 40).

### 4.2. Clinical Characteristics

**Figure 2:**
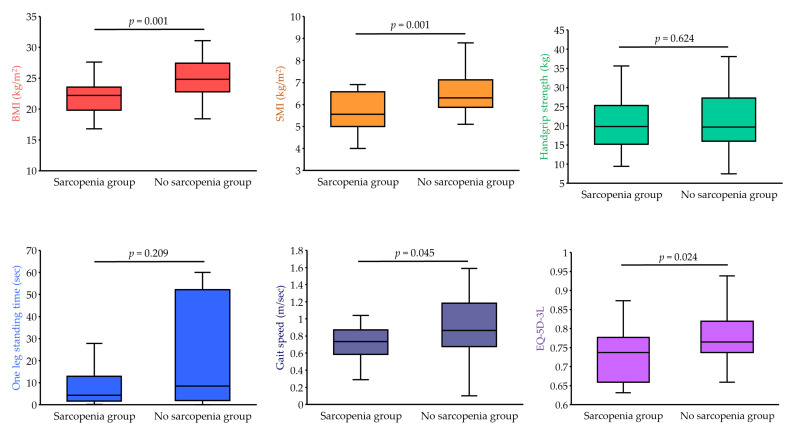
Comparison of body composition, physical function and HRQOL. BMI, body mass index; EQ-5D-3L, EuroQol 5-dimension 3-level questionnaire; SMI, skeletal muscle mass index.

Table 1 shows the characteristics of the older people with LTCI in the two groups. Compared to those in the no sarcopenia group, the older people in the sarcopenia group had significantly lower BMI, SMI, gait speed, and EQ-5D-3L (*p* < 0.05) (Figure 2).

### 4.3. HRQOL after Adjustment in the Two Groups

After adjusting for BMI, age, and sex, the sarcopenia group showed a significantly lower EQ-5D-3L (0.72 ± 0.01 vs. 0.78 ± 0.01, F = 14.7, *p* < 0.001) than that in the no sarcopenia group.

### 4.4. Correlation with HRQOL in the Two Groups

**Figure 3:**
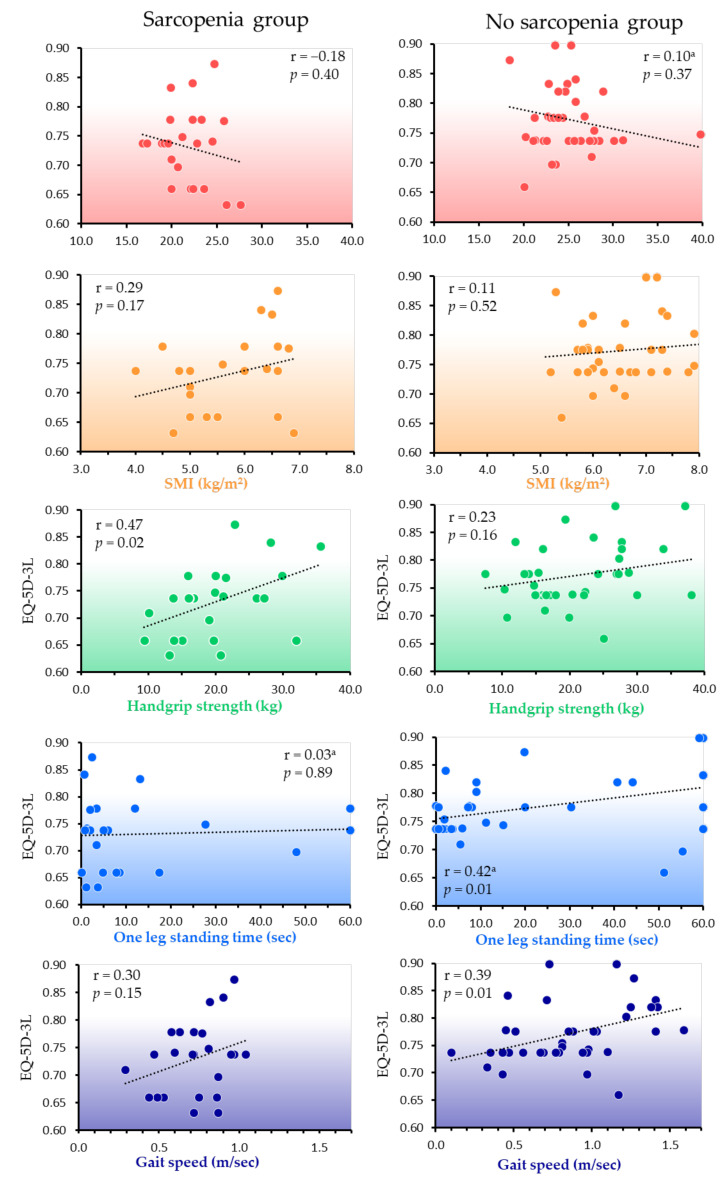
Correlation between HRQOL and body composition and physical function measures. Pearson’s correlation coefficient; ^a^ Spearman’s rank correlation coefficient. BMI, body mass index; EQ-5D-3L, EuroQol 5-dimension 3-level questionnaire; SMI, skeletal muscle mass index.

In the sarcopenia group, the EQ-5D-3L showed a significant correlation with handgrip strength (*p* = 0.02) and a moderate correlation coefficient. In the no sarcopenia group, the EQ-5D-3L showed a significant correlation with both gait speed (*p* = 0.01), with a moderate correlation coefficient, and with one-leg standing time (*p* = 0.01), but with a low correlation coefficient (Figure 3).

## 5. Discussion

To the best of our knowledge, this is the first report to show the difference in HRQOL of Japanese older people with and without sarcopenia covered by LTCI. Our results showed that older people with LTCI in the sarcopenia group had a lower HRQOL than those in the no sarcopenia group. HRQOL in the sarcopenia group was significantly correlated with handgrip strength (*p* = 0.02) and had a moderate correlation coefficient, whereas that in the no sarcopenia group was significantly correlated with both gait speed (*p* = 0.01), with a moderate correlation coefficient, and with one-leg standing time (*p* = 0.01), but with a low correlation coefficient.

### 5.1. Differences in HRQOL Related to Sarcopenia

The prevalence of sarcopenia determined using the Asian Working Group for Sarcopenia criteria 2019 is reported to be 5–19% among Japanese older people overall [10,11,12,13] and 48% for older people with LTCI [14]. Our results showed a prevalence of sarcopenia of 37.5%, which is slightly lower than that of the generalized population of older people with LTCI in the previous study [14]. In another previous study [41], the group with LTCI had a lower HRQOL than the group without LTCI [41]. In addition, the standard value of EQ-5D-3L is reported to be 0.8 or higher for those over 60 years old [42]. In this study among older people with LTCI, the value was low, i.e., less than 0.8, regardless of whether they did or did not have sarcopenia. This result may indicate serious problems with HRQOL in the older people with LTCI.

After adjusting for BMI, age, and sex when examining differences in HRQOL in those with and without sarcopenia, the EQ-5D-3L in the sarcopenia group was shown to be significantly lower than that in the no sarcopenia group. It is already known that sarcopenia in older people is associated with poor HRQOL [26]. Longitudinal studies also report that the presence of sarcopenia at baseline affects the long-term decline in HRQOL [43], and some reviews report a link between sarcopenia and HRQOL in older people [28,44]. In addition, multiple regression analysis results show that in middle-aged and older people, sarcopenia is an independent related factor for HRQOL [45]. Therefore, the presence of sarcopenia may be associated with low HRQOL, even in older people with LTCI.

### 5.2. Interrelationship with HRQOL in Those with and without Sarcopenia

The HRQOL in the sarcopenia group showed a moderately significant correlation with handgrip strength in this study. Previous studies have shown that handgrip strength is an indicator of prognosis, including mortality [46]. Low handgrip strength is associated with poor HRQOL [47], and severely ill patients with poor handgrip strength have a particularly low HRQOL [48]. In addition, the presence of sarcopenia in older people has been reported to affect HRQOL due to reduced handgrip strength [49]. Therefore, the decrease in HRQOL in the sarcopenia group, which is an indicator of severity, may be related to low handgrip strength.

The HRQOL of the no sarcopenia group showed a positive correlation between gait speed and one-leg standing time. Previous reports have shown that gait speed and balance are associated with maintaining HRQOL in older people [50,51]. Further, multiple regression analysis has shown gait speed and balance to be associated with improved physical HRQOL of older people [52]. Therefore, in older people with LTCI who do not have sarcopenia, gait speed and one-leg standing time may reflect their HRQOL.

### 5.3. Strengths and Limitations

Among older people with LTCI attending a daycare center in Japan, those with sarcopenia showed significantly lower HRQOL than those without sarcopenia. A significant correlation of HRQOL with handgrip strength was present in the sarcopenia group, whereas in the no sarcopenia group, HRQOL correlated significantly with both gait speed, with a moderate correlation coefficient, and with one-leg standing time, but with a low correlation coefficient. This may be important information for preventing the deterioration of and improving the HRQOL of older people with LTCI in both those with and without sarcopenia. That is, when providing routine care and support to these people, measures taken to increase handgrip strength when sarcopenia is present, and gait speed and one-leg standing time when sarcopenia is not present, may help maintain and improve their HRQOL. In the future, it will be important to accumulate data on the HRQOL of more participants to better clarify the relationship between sarcopenia and HRQOL.

This study has some limitations. It was conducted in a single facility, and the sample size is small. Therefore, it was not possible to classify and analyze the participants by sex. Due to the cross-sectional nature of this study, the causal relationship between sarcopenia and HRQOL could not be explained. Factors relating to sarcopenia such as nutrition, psychological function, and cognitive function could have an effect as confounding factors, but they were not investigated.

## 6. Conclusions

This study showed important differences in HRQOL in older people with and without sarcopenia covered by LTCI, especially in terms of their need for care and support. Among this study population, after adjusting for BMI, age, and sex, HRQOL was lower in those with sarcopenia than in those without sarcopenia. Furthermore, the HRQOL of the sarcopenia group was shown to be interrelated with handgrip strength, whereas that of the no sarcopenia group was interrelated with gait speed and one-leg standing time. This information may help to maintain and improve HRQOL in older people with and without sarcopenia covered by LTCI.

## Figures and Tables

**Table 1:** Characteristics of all participants and those of the sarcopenia and no sarcopenia groups.

	All Participants *n* = 64	Sarcopenia *n* = 24	No Sarcopenia *n* = 40	t, Z or χ^2^ Value	*p* Value
Age, years	79.3 ± 8.8	79.4 ± 9.8	79.2 ± 8.3	−0.1	0.940
Sex, male, %	32.8	45.8	25.0	2.9 ^b^	0.086
BMI, kg/m^2^	23.8 ± 3.7	21.9 ± 2.8	25.0 ± 3.8	3.4 ^a^	0.001
SMI, kg/m^2^	6.2 ± 0.9	5.7 ± 0.9	6.5 ± 0.8	3.6	0.001
LTCI level, support Level 1/2/care Level 1/2/3, %	45.3/21.9/20.3/9.4/3.1	45.8/25.0/12.5/16.7/0	45.0/20.0/25.0/5.0/5.0	4.7 ^b^	0.319
Comorbidities, %					
Hypertension	68.8	70.8	67.5	0.1 ^b^	0.781
Diabetes	21.9	29.2	17.5	1.2 ^b^	0.274
Orthopedic disease	53.1	50.0	55.0	0.2 ^b^	0.698
Neurological disease	46.9	54.2	42.5	0.8 ^b^	0.365
Cardiovascular disease	76.5	78.3	74.4	0.1 ^b^	0.729
Cancer disease	10.9	8.3	12.5	0.3 ^b^	0.605
Physical function					
Handgrip strength, kg	20.8 ± 7.4	20.1 ± 6.8	21.1 ± 7.7	0.5	0.624
One-leg standing time, sec	18.3 ± 22.5	12.3 ± 18.2	22.1 ± 24.3	1.2 ^a^	0.209
Gait speed, m/sec	0.82 ± 0.31	0.73 ± 0.19	0.87 ± 0.36	2.1	0.045
EQ-5D-3L	0.76 ± 0.06	0.73 ± 0.07	0.77 ± 0.06	2.2 ^a^	0.024

Values are presented as mean ± standard deviation or %, ^a^: Z value, ^b^: χ^2^ value. BMI, body mass index; EQ-5D-3L, EuroQol 5-dimension 3-level questionnaire; LTCI, long-term care insurance; SMI, skeletal muscle mass index.

## Data Availability

Not applicable.

## References

[1] United Nations The 2019 Revision of World Population Prospects. https://esa.un.org/unpd/wpp/.

[2] Statistics Bureau Ministry of Internal Affairs and Communications. https://www8.cao.go.jp/kourei/whitepaper/w-2021/gaiyou/pdf/1s1s.pdf.

[3] Campbell J.C., Ikegami N. (2000). Long-term care insurance comes to Japan. Health Aff..

[4] Tamiya N., Noguchi H., Nishi A., Reich M.R., Ikegami N., Hashimoto H., Shibuya K., Kawachi I., Campbell J.C. (2011). Population ageing and wellbeing: Lessons from Japan’s long-term care insurance policy. Lancet.

[5] Ministry of Health, Labour and Welfare Long-Term Care, Health and Welfare Services for the Elderly. https://www.mhlw.go.jp/english/policy/care-welfare/care-welfare-elderly/index.html.

[6] Outline of Long-Term Care Insurance Business Status Report Ministry of Health, Labor and Welfare. https://www.mhlw.go.jp/topics/kaigo/osirase/jigyo/m21/dl/2112a.pdf.

[7] Situation Surrounding the Long-Term Care Field Ministry of Health, Labor and Welfare. https://www.mhlw.go.jp/content/12300000/000608284.pdf.

[8] Ikegami N. (2019). Financing long-term care: Lessons from Japan. Int. J. Health Policy Manag..

[9] Inoue N. (2012). The chronological trend of the bedridden status and preventative factors and cumulative survival rate during three years in the Japanese urban elderly dwellers. Bull. Soc. Med..

[10] Shimada H., Tsutsumimoto K., Doi T., Lee S., Bae S., Nakakubo S., Makino K., Arai H. (2021). Effect of sarcopenia status on disability incidence among Japanese older adults. J. Am. Med. Dir. Assoc..

[11] Chen L.K., Woo J., Assantachai P., Auyeung T.W., Chou M.Y., Iijima K., Jang H.C., Kang L., Kim M., Kim S. (2020). Asian Working Group for Sarcopenia: 2019 Consensus Update on Sar-copenia Diagnosis and Treatment. J. Am. Med. Dir. Assoc..

[12] Kitamura A., Seino S., Abe T., Nofuji Y., Yokoyama Y., Amano H., Nishi M., Taniguchi Y., Narita M., Fujiwara Y. (2021). Sarcopenia: Prevalence, associated factors, and the risk of mortality and disability in Japanese older adults. J. Cachexia Sarcopenia Muscle.

[13] Kugimiya Y., Iwasaki M., Ohara Y., Motokawa K., Edahiro A., Shirobe M., Watanabe Y., Obuchi S., Kawai H., Fujiwara Y. (2021). Relationship between oral hypofunction and sarcopenia in community-dwelling older adults: The Otassha Study. Int. J. Environ. Res. Public Health.

[14] Sato R., Sawaya Y., Shiba T., Hirose T. (2020). Relationship between depression and sarcopenia in Japanese elderly with mild long-term care or support needs. Rigakuryoho Kagaku.

[15] Marincolo J.C.S., Aprahamian I., Corona L.P., Neri A.L., Yassuda M.S., Borim F.S.A. (2021). Three definitions of probable sarcopenia and associations with falls and functional disability among community-dwelling older adults. Osteoporos. Sarcopenia.

[16] Beaudart C., Zaaria M., Pasleau F., Reginster J.Y., Bruyère O. (2017). Health outcomes of sarcopenia: A systematic review and meta-analysis. PLoS ONE.

[17] Lin P.-C., Chang S.-F., Ho H.-Y. (2020). Effect of whole-body vibration training on the physical capability, activities of daily living, and sleep quality of older people with sarcopenia. Appl. Sci..

[18] Kitamura M., Izawa K.P., Ishihara K., Matsuda H., Okamura S., Fujioka K. (2021). Physical activity and sarcopenia in community-dwelling older adults with long-term care insurance. Eur. J. Investig. Health Psychol. Educ..

[19] World Health Organization (2002). Active ageing: A policy framework. Aging Male.

[20] Ikegami S., Takahashi J., Uehara M., Tokida R., Nishimura H., Sakai A., Kato H. (2019). Physical performance reflects cognitive function, fall risk, and quality of life in community-dwelling older people. Sci. Rep..

[21] Crocker T.F., Brown L., Clegg A., Farley K., Franklin M., Simpkins S., Young J. (2019). Quality of life is substantially worse for community-dwelling older people living with frailty: Systematic review and meta-analysis. Qual. Life Res..

[22] Iijima K., Arai H., Akishita M., Endo T., Ogasawara K., Kashihara N., Hayashi Y.K., Yumura W., Yokode M., Ouchi Y. (2021). Toward the development of a vibrant, super-aged society: The future of medicine and society in Japan. Geriatr. Gerontol. Int..

[23] Damayanthi H.D.W.T., Moy F.M., Abdullah K.L., Dharmaratne S.D. (2018). Health related quality of life and its associated factors among community-dwelling older people in Sri Lanka: A cross-sectional study. Arch. Gerontol. Geriatr..

[24] Verlaan S., Aspray T.J., Bauer J.M., Cederholm T., Hemsworth J., Hill T.R., McPhee J.S., Piasecki M., Seal C., Sieber C.C. (2017). Nutritional status, body composition, and quality of life in community-dwelling sarcopenic and non-sarcopenic older adults: A case-control study. Clin. Nutr..

[25] Sun S., Lee H., Yim H.W., Won H.S., Ko Y.H. (2019). The impact of sarcopenia on health-related quality of life in elderly people: Korean National Health and Nutrition Examination Survey. Korean J. Intern. Med..

[26] Manrique-Espinoza B., Salinas-Rodríguez A., Rosas-Carrasco O., Gutiérrez-Robledo L.M., Avila-Funes J.A. (2017). Sarcopenia is associated with physical and mental components of health-related quality of life in older adults. J. Am. Med. Dir. Assoc..

[27] Kim S., Kim M., Won C.W. (2018). Validation of the Korean Version of the SARC-F Questionnaire to Assess Sarcopenia: Korean Frailty and Aging Cohort Study. J. Am. Med. Dir. Assoc..

[28] Woo T., Yu S., Visvanathan R. (2016). Systematic literature review on the relationship between biomarkers of sarcopenia and quality of life in older people. J. Frailty Aging.

[29] Chiba I., Lee S., Bae S., Makino K., Shinkai Y., Katayama O., Harada K., Takayanagi N., Shimada H. (2021). Difference in sarcopenia characteristics associated with physical activity and disability incidences in older adults. J. Cachexia Sarcopenia Muscle.

[30] Kitamura M., Izawa K.P., Ishihara K., Matsuda H., Okamura S., Fujioka K. (2021). Prevalence and related factors of sarcopenia in community-dwelling elderly with long-term care insurance. Rev. Recent Clin. Trials.

[31] Uemura K., Doi T., Lee S., Shimada H. (2019). Sarcopenia and low serum albumin level synergistically increase the risk of incident disability in older adults. J. Am. Med. Dir. Assoc..

[32] Imai H., Fujii Y., Fukuda Y., Nakao H., Yahata Y. (2008). Health-related quality of life and beneficiaries of long-term care insurance in Japan. Health Policy.

[33] Saito T., Izawa K.P., Matsui N., Arai K., Ando M., Morimoto K., Fujita N., Takahashi Y., Kawazoe M., Watanabe S. (2017). Comparison of the measurement properties of the Functional Independence and Difficulty Scale with the Barthel Index in community-dwelling elderly people in Japan. Aging Clin. Exp. Res..

[34] Hsieh C.T., Yamazaki H., Wang J., Kamitani T., Yamamoto Y., Fukuhara S. (2021). Quality of life and disability-free survival in the elderly: The Locomotive Syndrome and Health Outcome in Aizu Cohort Study. J. Aging Health.

[35] Baumgartner R.N., Koehler K.M., Gallagher D., Romero L., Heymsfield S.B., Ross R.R., Garry P.J., Lindeman R.D. (1998). Epi-demiology of sarcopenia among the elderly in New Mexico. Am. J. Epidemiol..

[36] Ida S., Kaneko R., Murata K. (2018). SARC-F for screening of sarcopenia among older adults: A meta-analysis of screening test ac-curacy. J. Am. Med. Dir. Assoc..

[37] Tanaka M., Ikezoe T., Ichihashi N., Tabara Y., Nakayama T., Takahashi Y., Matsuda F., Tsuboyama T., Nagahama Study Group (2020). Relationship of low muscle mass and obesity with physical function in community dwelling older adults: Results from the Nagahama study. Arch. Gerontol. Geriatr..

[38] Japanese EuroQol Translation Team (1998). The development of Japanese EuroQol instrument. Iryo Shakai.

[39] Tsuchiya A., Ikeda S., Ikegami N., Nishimura S., Sakai I., Fukuda T., Hamashima C., Hisashige A., Tamura M. (2002). Estimating an EQ-5D population value set: The case of Japan. Health Econ..

[40] Guilford J.P. (1956). Fundamental Statistics in Psychology and Education.

[41] Chen W., Fukutomi E., Wada T., Ishimoto Y., Kimura Y., Kasahara Y., Sakamoto R., Okumiya K., Matsubayashi K. (2013). Comprehensive geriatric functional analysis of elderly populations in four categories of the long-term care insurance system in a rural, depopulated and aging town in Japan. Geriatr. Gerontol. Int..

[42] Shiroiwa T., Fukuda T., Ikeda S., Igarashi A., Noto S., Saito S., Shimozuma K. (2016). Japanese population norms for preference-based measures: EQ-5D-3L, EQ-5D-5L, and SF-6D. Qual. Life Res..

[43] Veronese N., Koyanagi A., Cereda E., Maggi S., Barbagallo M., Dominguez L.J., Smith L. (2022). Sarcopenia reduces quality of life in the long-term: Longitudinal analyses from the English longitudinal study of ageing. Eur. Geriatr. Med..

[44] Tsekoura M., Kastrinis A., Katsoulaki M., Billis E., Gliatis J. (2017). Sarcopenia and its impact on quality of life. Adv. Exp. Med. Biol..

[45] Fábrega-Cuadros R., Hita-Contreras F., Martínez-Amat A., Jiménez-García J.D., Achalandabaso-Ochoa A., Lavilla-Lerma L., García-Garro P.A., Álvarez-Salvago F., Aibar-Almazán A. (2021). Associations between the severity of sarcopenia and health-related quality of life in community-dwelling middle-aged and older adults. Int. J. Environ. Res. Public Health.

[46] Wu Y., Wang W., Liu T., Zhang D. (2017). Association of grip strength with risk of all-cause mortality, cardiovascular diseases, and cancer in community-dwelling populations: A meta-analysis of prospective cohort studies. J. Am. Med. Dir. Assoc..

[47] Kang S.Y., Lim J., Park H.S. (2018). Relationship between low handgrip strength and quality of life in Korean men and women. Qual. Life Res..

[48] Kim H., Yoo S., Kim H., Park S.G., Son M. (2021). Cancer survivors with low hand grip strength have decreased quality of life compared with healthy controls: The Korea National Health and Nutrition Examination Survey 2014–2017. Korean J. Fam. Med..

[49] Marques L.P., Confortin S.C., Ono L.M., Barbosa A.R., d’Orsi E. (2019). Quality of life associated with handgrip strength and sarcopenia: EpiFloripa Aging Study. Arch. Gerontol. Geriatr..

[50] Steckhan G.M.A., Fleig L., Schwarzer R., Warner L.M. (2022). Perceived physical functioning and gait speed as mediators in the association between fear of falling and quality of life in old age. J. Appl. Gerontol..

[51] Davis J.C., Bryan S., Best J.R., Li L.C., Hsu C.L., Gomez C., Vertes K.A., Liu-Ambrose T. (2015). Mobility predicts change in older adults’ health-related quality of life: Evidence from a Vancouver falls prevention prospective cohort study. Health Qual. Life. Outcomes.

[52] Taani M.H., Strath S.J., Cho C.C., Ellis J., Oh H. (2022). Objective physical activity levels, sedentary time, and muscle mass, strength, and function: Impact on physical and mental health-related quality of life in older adults. Res. Gerontol. Nurs..

